# The Changing Epidemiology of Cystic Fibrosis: Incidence, Survival and Impact of the *CFTR* Gene Discovery

**DOI:** 10.3390/genes11060589

**Published:** 2020-05-26

**Authors:** Virginie Scotet, Carine L’Hostis, Claude Férec

**Affiliations:** 1 Inserm, University of Brest, EFS, UMR 1078, GGB, F-29200 Brest, France; carine_lhostis@hotmail.com (C.L.); claude.ferec@univ-brest.fr (C.F.); 2 Department of Molecular Genetics, University Hospital of Brest, F-29200 Brest, France

**Keywords:** cystic fibrosis, *CFTR* gene, incidence, survival, genotype-phenotype correlations, health policies, newborn screening, CFTR modulators

## Abstract

Significant advances in the management of cystic fibrosis (CF) in recent decades have dramatically changed the epidemiology and prognosis of this serious disease, which is no longer an exclusively pediatric disease. This paper aims to review the changes in the incidence and survival of CF and to assess the impact of the discovery of the responsible gene (the *CFTR* gene) on these changes. The incidence of CF appears to be decreasing in most countries and patient survival, which can be monitored by various indicators, has improved substantially, with an estimated median age of survival of approximately50 years today. Cloning of the *CFTR* gene 30 years ago and efforts to identify its many mutations have greatly improved the management of CF. Implementation of genetic screening policies has enabled earlier diagnosis (via newborn screening), in addition to prevention within families or in the general population in some areas (via prenatal diagnosis, family testing or population carrier screening). In the past decade, in-depth knowledge of the molecular bases of CF has also enabled the emergence of CFTR modulator therapies which have led to major clinical advances in the treatment of CF. All of these phenomena have contributed to changing the face of CF. The advent of targeted therapies has paved the way for precision medicine and is expected to further improve survival in the coming years.

## 1. Introduction

Cystic fibrosis (CF) has traditionally been defined as the most common life-threatening inherited disorder of children in Caucasian populations, with an incidence of 1/2500 live births [1]. This definition is no longer appropriate today. Although CF remains a serious disorder, advances in the treatment and management of the disease have remarkably changed the characteristics of the CF population [2,3,4,5,6,7].

Epidemiological changes have occurred both in the incidence of CF, which seems to be decreasing in most countries, and in the survival of CF patients, which has greatly improved in recent decades [4,5,6,7,8]. When CF was first described by Dorothy H. Anderson in 1938 [9], the patients usually died in their first year of life. Nowadays, the proportion of adult patients exceeds that of children in developed countries and the estimated median age of survival is close to 50 years [10,11,12], which means that half of the babies born today with CF may expect to survive into their fifth decade. From an exclusively pediatric disease, CF has gradually also become a disease of the adult, with new associated pathologies to be managed. Such epidemiological changes can be tracked by reliable tools such as CF patient registries, which monitor the demographical and clinical characteristics of the CF population.

The discovery of the gene responsible for CF—the *CFTR* gene—30 years ago [13,14,15] marked an important milestone in the history of CF. It has upset our knowledge of the pathophysiology of the disease, contributed to improving the diagnosis and treatment of CF patients and paved the way for novel therapeutic approaches and the advent of targeted therapies [16]. This discovery has contributed and will continue to contribute to the epidemiological changes observed in CF, through the implementation of genetic-based health policies that allow early diagnosis or prevention within families and/or populations, and the emergence of CFTR modulator therapies [17].

This paper reviews the changes that have occurred in the epidemiology of CF, focusing on incidence and survival, and reports the impact of the discovery of the *CFTR* gene on these changes.

## 2. The Incidence of Cystic Fibrosis

### 2.1. Estimates of the Incidence of CF Worldwide

The incidence of CF has traditionally been estimated at 1/2500 live births in a populations of European descent [1]. However, data from newborn screening (NBS) programs for CF reveal that the incidence appears to be lower than in the past. Today, the incidence of CF is estimated, on average, between 1/3000 and 1/6000 in such populations [18,19], which corresponds to carrier rates of 1/28 and 1/40, respectively.

Previously, the incidence of CF was generally estimated from epidemiological studies, which may have been biased by under diagnosis and/or underreporting of cases. Incidence estimates have become more reliable since the implementation of NBS for CF, which has rapidly expanded worldwide in the last decade [19]. The complete registration of cases at birth has led to an accurate measurement of the incidence and better monitoring of its time trends. Nevertheless, even with NBS data, it remains of upmost importance to ensure that studies include the same type of patients when comparing incidence data between countries (such as inclusion or not of false negatives and of patients with meconium ileus).

In a very recent paper describing why NBS for CF is worthwhile, we reviewed the latest data on the incidence of CF worldwide [19]. In Europe, the incidence ranges from 1/1353 in Ireland [20] to 1/25,000 in Finland [21] and is on average 1/4500 in Western Europe [22,23,24] and 1/6000 in Northern and Central Europe [25,26,27]. In Australasia, where a NBS program has been implemented for a long time, the incidence is well established and is on average 1/3000 [28]. The incidence is also approximately1/3300 in Canada [29] and 1/4000 in the USA, where large ethnical variations are observed [30,31].

Very high incidences of CF, probably due to genetic drift and founding effects, have been reported in small isolated populations, such as in the Amish population in Ohio (1/569) [32] or in Saguenay-Lac-Saint-Jean in Quebec (1/902) [33].

The disease is much rarer in other parts of the world. In Latin and South America, the incidence of CF remains difficult to estimate in most countries due to the lack of registries and NBS programs as well as the high ethnic admixture of the population. The average incidence seems to be approximately1/8000 to 1/10,000, ranging from 1/6100 in Argentina to 1/15,000 in Costa Rica [19]. In Asian populations, the existence of CF is now better established, but the incidence remains underestimated in most countries. It appears higher in the Middle East (where consanguinity is common) than in East Asia. The incidence ranges from 1/2560 in Jordan [34] to 1/350,000 in Japan [35] and is estimated between 1/10,000 and 1/100,000 in the Indian population [36,37]. Few data are available in African populations [38].

### 2.2. Time Trends in the Incidence of CF

Time trends in the incidence of CF have been investigated in several studies, most of which reported a decline [23,39,40,41,42,43] but not all [44,45]. This was the case for example in two American states: in Colorado which observed no decline in incidence over a 24 year period (1983–2006) [44] and in Wisconsin, which analyzed time trends over an 18 year period (1994–2011) and even observed a trending (but not significant) increase in incidence in recent years (in different genotype groups and in all ethnic groups) [45]. In Brittany (western France), we analyzed time trends in the incidence of CF over a 35 year period and observed a significant decline (from 1/1983 in the late 1970s to 1/3268 over the 2005–2009 period) but also a clear breakpoint at the end of the 1980s, which seemed consecutive to the availability of prenatal diagnosis [42].

The temporal trends observed in incidence result from the combination of many factors. They stem from demographic changes (such as larger population admixtures, decreasing consanguinity, and decreasing fertility rates), from implementation of genetic-based health policies allowing prevention within families or populations (such as prenatal diagnosis, genetic preimplantation diagnosis, family testing, prenatal screening, and population carrier screening), but also from cultural behaviors toward use of genetic testing, prenatal diagnosis and pregnancy terminations. The causes of the changes observed in incidence therefore vary by region and by population. Our team has shown that CF health policies implemented in Brittany have reduced the incidence of CF by approximately one-third over the study period [42,46].

The greatest declines in the incidence of CF have been observed in areas where prenatal screening or population carrier screening (aiming at identifying all the couples with a one-in-four risk of having a CF child in a population) is underway [23,39,40,43]. For example, in Massachusetts (USA), where NBS for CF was implemented in 1999, Hale et al. reported a significant decrease in the number of screened CF patients since the publication of the US recommendations for population carrier screening in 2003 [39]. The authors also observed a change in the structure of the screened cohort, with a lower proportion of p.Phe508del homozygous patients. An Italian study, which analyzed time trends in the incidence of CF in the Veneto/Trentino Alto Adige area over a 21 year period (1993–2013), also observed a significant decline in incidence that was higher (~35%) in the eastern part of the area where population carrier screening is underway [23,40]. Similarly, Stafler et al. analyzed the impact of population carrier screening on the incidence of CF in Israel [43]. This country, which has not set up a NBS program for CF but which initiated population carrier screening in 1999, observed a marked decline in the incidence of CF (~60%) over a 22 year period.

However, comparison of time trends in incidence between areas remains complex due to variability in study periods but also in public health policies that are implemented in those areas.

## 3. The Survival of Cystic Fibrosis Patients

### 3.1. The Changing Face of CF

Prognosis of CF patients has greatly improved in recent decades. One of the most striking evidences of this change is the substantial growth in the proportion of adult patients, which currently exceeds 50% in most countries, and even 60% in Canada (Table 1) [10,11,12,47,48,49,50,51]. In this country, the proportion of adult patients has more than doubled in 35 years, increasing from 29.5% in 1984 to 61.5% in 2018 [12]. This growth should continue, as illustrated by a study based on the European CF Registry, which predicted that the number of adult patients living with CF in Europe was expected to increase by 75% between 2010 and 2025 [52].

Cystic fibrosis, long qualified as a pediatric disease, has also gradually become a disease of the adult. The transition to adult care center is now a key step for patients and their family, and new elements have to be taken into account in disease management, such as employability, desire for married life, and parenthood [53]. The number of pregnancies and paternities identified in CF patients has therefore progressively increased and one of the challenges is now to assess the safety of CFTR modulator therapies in pregnancy and breastfeeding [54,55]. As a result of the changing epidemiology, a growing number of studies has also been devoted to patients who reach the age of 40 [56,57]. These “long survivors” now represent 11.9% of the CF population in France and 15.9% in Canada (Table 1). This population, which includes patients carrying mild genotypes but also patients diagnosed in adulthood, is particularly interesting for identifying the predictors of better survival [58].

Many factors are responsible for these major advances such as standardization of care, with management of patients in specialized centers by multidisciplinary teams, better control of pulmonary infection with the development of new inhaled therapies, better control of *Pseudomonas aeruginosa* colonization, aggressive nutritional supplementation with pancreatic enzymes, early diagnosis through newborn screening, and lung transplantation [5].

Providing up-to-date estimates of survival is crucial for advising patients and their families on life expectancy, planning health care needs and guiding the development of new therapies. 

### 3.2. Better Understanding of Survival Indicators

For many years, changes in prognosis of CF were described by monitoring the proportion of adult patients, the death rates or the median age at death. This last metric, which informs on the distribution of the age of patients at time of their death, is not a survival metric as it does not consider the patients who survived.

Nowadays, time trends in survival can be monitored by additional metrics such as survival probabilities by birth cohort, the estimated median age of survival(also called the predicted median survival age) and the estimated median age of survival conditional on living to a given age (see below). It should be noted that life expectancy, which is often misused in CF, is almost never estimated.

There are several ways to estimate survival in CF and the terminology used in that field is complex and often confusing for patients and their families, but also for the medical community. It is, however, crucial for clinicians to understand the difference between the various metrics, so that they can provide appropriate information to patients. A very comprehensive guide has recently been published by Keogh and Stanojevic [59] in order to facilitate the interpretation of the estimated median age of survival in CF and to standardize the presentation of survival data in CF patient registry reports. One other paper by Sykes et al. explains very well the three methods for estimating survival [60]. Briefly, these methods are:

(1) The birth cohort approach, which is a longitudinal method that consists of following one or several birth cohorts and registering all the deaths that occur in those cohorts over time. This method, which requires time, draws for each birth cohort a Kaplan–Meier survival curve, which looks like a staircase curve that goes down at each death. This enables determination of the median survival when 50% of the patients of the cohort have died.
(2) The period approach, which is a cross-sectional method that is commonly used by registries. It analyzes the structure of the CF population present in a registry on a specified period (usually a 5 year window; for example, the period 2014–2018) and estimates a survival curve by applying the age-specific mortality rates observed among those prevalent cases to a fictive cohort. This method estimates the median age of survival from birth, which corresponds to the age beyond which half of the babies born today with CF are expected to live. This approach assumes that death rates remain unchanged over time (which is not true) and requires large samples.
(3) The conditional survival approach, which was applied recently in CF [61]. As the estimated median age of survival only applies to babies born today and as some patients have already surpassed the estimated age, another metric has been proposed recently: the estimated median age of survival conditional to surviving to a given age (for example, age of 30 or 40). This metric represents the age at which 50% of the patients who have already survived to the given age are expected to live. It is more relevant for CF patients and is higher than the estimate from birth. Keogh et al. showed, using data from the UK CF Registry, that in p.Phe508del homozygous patients, the estimated median age of survival from birth was 46 years in males and 41 years in females, whereas the estimated median age of survival conditional on surviving to 30 was 6 and 8 years higher, respectively [61].

The prerequisite for a quality survival analysis is to have a well-defined study population with complete registration of CF cases and deaths. Comparisons of survival data between countries require standardization in data processing and analysis.

### 3.3. Current Survival Estimates in CF

National CF registries are valuable tools for performing quality survival analyzes and have been instrumental in demonstrating improved survival. Examination of the annual CF registry reports shows, however, that the presentation of survival data is not homogeneous [10,11,12,47,48,49,50,51]. To date, all CF registries show the median age at death, which may be supplemented by a graph representing the distribution of ages at death or the time trends in this median age at death. Four registries (the Canadian, Irish, UK and US ones) determine the estimated median age of survival based on the period approach. The French and Canadian registries show survival probabilities by birth cohort, while the US registry also presents two other metrics: a graph representing the estimates of conditional survival at specific ages (up to 40 years) and another one illustrating time trends in annual mortality rates.

Longitudinal monitoring of registry data shows that the median age at death and the estimated median age of survival continue to increase gradually, while mortality rates decrease. When CF was described for the first time in 1938 [9], patients with CF usually died in their first year of life. In the 1960s, they rarely survived beyond the age of five. The most recent CF registry data (2017 or 2018 annual reports) show that the median age at death ranges from 29.0 years (ECFS registry) to 35.6 years (Australian registry) (Table 1). The estimated median age of survival, which is determined by four registries, is currently44.4 years in Ireland, 47.3 years in the UK, 47.4 years in the USA and 52.1 years in Canada (Table 1). This metrics has increased by more than 15 years in 30 years in the USA [12]. Regarding conditional survival analysis, the US registry report shows that the estimated median age of survival is close to 55 years for patients who reach the age of 30, and exceeds 60 years for patients who reach the age of 40 [12].

### 3.4. Prognostic Factors 

Although survival estimate has greatly improved globally, it continues to be impacted by various individual factors. Beyond the main predictor of worse survival that is lung function (FEV_1_ < 30% predicted) [62], other factors have been associated with reduced survival such as female sex, higher age at diagnosis, severe *CFTR* genotype, ethnic background, lower socio-economic status, worse nutritional status, pancreatic insufficiency, early colonization with *Pseudomonas aeruginosa*, and presence of diabetes [5]. Prognostic scores based on various variables have thus been developed to predict the risk of death or the risk of lung transplantation [63,64,65,66]. 

Two other factors should have a major impact on the survival of CF, which will have to be measured precisely in the coming years: the expanding implementation of NBS for CF and the emergence of targeted therapies.

### 3.5. New Statistical Developments and Future Trends in Survival

A survey that was recently carried out to decipher how CF patients access and use information on life expectancy shows that most respondents wanted more personalized survival data [67]. Mathematical developments are therefore underway in order to further improve the modeling of survival by providing personalized estimates. One of the challenges is to develop models based on longitudinal data that are able to consider the current health status of the patients (and not only their baseline characteristics). A dynamic predictive model providing personalized estimates of survival was recently developed using data from the UK CF Registry [68]. This model, which integrates 16 predictors, is able to predict survival up to 10 years for patients up to 50 years of age.

The estimated median age of survival of CF patients, which is close to 50 years today, is expected to continue to improve in the future, with the rapid expanding of NBS for CF worldwide over the past decade and with the recent advent of CFTR modulator therapies. Further work is needed to assess the effect of these factors on the survival of CF. Some studies have suggested that NBS for CF results in a prolonged survival, but few are yet able to assess its long-term effects. The studies performed in that field are presented in the next section.

The increase in survival estimates undeniably changes the population’s perception of the disease and leads to ethical reflection on the decisions to be made by couples when CF is diagnosed before birth. 

## 4. The Impact of the Discovery of the *CFTR* Gene on the Epidemiology of the Disease

The discovery of the *CFTR* gene in 1989 marked an important milestone in the history of CF and raised tremendous hope in the medical and scientific community. An exemplary collaboration involving more than 100 laboratories worldwide and carried out through an international consortium for the study of the gene mutations (Cystic Fibrosis Genetic Analysis Consortium) has led to the identification of more than 2000 different *CFTR* mutations to date [69]. The molecular exploration of this gene has enabled a better understanding of the genotype/phenotype correlations, improved the diagnosis and the management of CF patients and their families, and opened up the way to the emergence of mutation-specific therapies, which contribute to modify the epidemiology of CF [16,17]. In a very comprehensive article published in this special issue of *Genes*, Farrell et al. reviews the “impact of the *CFTR* gene discovery on CF diagnosis, genetic counseling and preventive therapy” [16].

### 4.1. Study of Genotype/Phenotype Correlations

The growing number of mutations identified in the *CFTR* gene and the variability observed in the phenotypic expression of CF have led the researchers to try to establish genotype/phenotype correlations [70,71].

Quickly after the gene discovery, the *CFTR* mutations could be classified into six classes according to their impact on the level of protein function [72]. Schematically, mutations in classes I, II, and III are usually associated with a classical form of CF (severe mutations), while those in classes IV, V, and VI are related to a milder phenotype (mild mutations) characterized by pancreatic sufficiency and later bacterial colonization. The estimated median age of survival of patients carrying at least one mild mutation is generally ten years higher than that of patients with severe mutations.

Various tools are available to help identify the clinical impact of a *CFTR* variant: (1) international mutations databases such as the Cystic Fibrosis Mutation Database (http://www.genet.sickkids.on.ca/), CFTR2 database (https://cftr2.org/) or UMD-CFTR database (http://www.umd.be/CFTR/); (2) CF registries, which may assist genetic counseling by providing aggregated clinical data associated with a given genotype; (3) bioinformatics prediction tools such as Polyphen or SIFT applications.

Despite these tools, it often remains difficult to predict with certainty the phenotype of a given genotype, and it quickly became obvious that the *CFTR* genotype could not explain all the phenotypic variability observed in CF, in particular in lung damage. Further research has been undertaken to identify other factors that influence the severity of the disease, including gene modifiers (such as genes involved in the immune response or in inflammation) [73,74], epigenetic factors and environmental factors (such as tobacco, pollution, socio-economic status, and adherence to therapies) [75].

The study of genotype/phenotype correlations also highlighted the existence of conditions associated with CFTR dysfunction that do not fulfill diagnostic criteria for CF. Those disorders, called *CFTR*-related disorders (*CFTR*-RDs) [76], include congenital bilateral absence of vas deferens, acute recurrent or chronic pancreatitis and disseminated bronchiectasis. Inclusion of patients with such conditions in epidemiological studies dealing with incidence or survival of CF can bias the estimates and the time trends.

### 4.2. Implementation of Genetic-Based Health Policies

In-depth knowledge of the molecular abnormalities of the *CFTR* gene [77] has enabled genetic screening policies to be implemented, allowing prevention within families (such as prenatal diagnosis for one-in-four at-risk couples or carrier testing in families) or in the general population in some areas (such as population carrier screening). As mentioned above, these health policies have contributed to reducing—in most countries and with varying degrees of importance—the incidence of CF through the identification of cases before birth [23,39,40,41,42,43,46].

The discovery of the *CFTR* gene has also increased the performance of NBS for CF by introducing DNA analysis into the screening protocol, and thus improved the diagnosis and management of CF [16,78,79]. The coupling of the immunereactive trypsinogen assay to the search for *CFTR* mutations has eliminated the need for a second blood sample. This has reduced the anxiety generated in families, leading to an earlier diagnosis and early access to specialized care centers, which prevents malnutrition and lung damage [80]. The positive effects of NBS for CF on short-term and long-term clinical outcomes are widely recognized [80,81,82,83] and include better nutritional status, lower pancreatic insufficiency, better lung function, lower infection rates, fewer and shorter hospitalizations. Some studies have shown that NBS for CF results in prolonged survival [84,85,86], but it is still too early for most countries to assess its long-term impact on survival. This could recently be measured in an Italian area in which NBS for CF has been running for over 40 years [87]. Tridello et al. reported a significantly higher survival probability at 20 years in the screened than in the non-screened group, both in patients with severe (84.9% vs. 63.6%; difference: 21.3%; *p* = 0.007) and moderate disease (94.5% vs. 85.9%; difference: 8.6%; *p* = 0.016). A 9% difference was also observed in the survival probability at 30 years (80.1% vs. 71.0%) but was not significant [87]. Through early diagnosis, NBS for CF maximizes survival in severe CF. The expanding of NBS worldwide in the past decade will inevitably continue to impact survival in the future.

### 4.3. Advent of CFTR Modulator Therapies

Deciphering of the molecular bases of CF has also led to the development of novel therapeutic approaches and the search for pharmaceutical treatments aiming at correcting the defective CFTR protein. These drugs, called CFTR modulators, search to improve the production, processing or expression of the protein and include correctors, potentiators, stabilizers, amplifiers and read through agents [88,89]. This approach is said to be “targeted” or “mutation specific” because the type of molecules to be administered to patients depends on the type of *CFTR* mutations they carry. Many studies have been carried out in that field over the past decade and have led to major clinical advances in treatment, with significant improvements in biological and clinical endpoints of CF (as sweat chloride concentration orFEV_1_) [90]. 

It was almost 25 years after the discovery of the *CFTR* gene that the first CFTR modulator (ivacaftor—Kalydeco^®^) could be marketed. This potentialtor was approved in 2012 for the treatment of CF patients aged ≥6 years carrying at least one G551D mutation [91]. This treatment was associated with significant improvements at day 28 in sweat chloride level, nasal potential difference and lung function (median increase of 8.7 points in the percentage of predicted FEV_1_). In 2015, the combination of ivacaftor with a corrector (lumacaftor/ivacaftor—Orkambi^®^) was then approved for patients aged ≥12 years who were p.Phe508del homozygous [92]. This combination significantly increased the percentage of predicted FEV_1_ (from 2.6 to 4.0 points) and was associated with a lower rate of pulmonary exacerbations, hospitalizations and use of intravenous antibiotics. In 2018, a second dual combination (tezacaftor/ivacaftor—Symdeko^®^) appeared [93] and, very recently, a triple combination therapy (elexacaftor/tezacaftor/ivacaftor–Trikafta™) was approved for the treatment of patients aged ≥12 years carrying at least one p.Phe508del mutation [94]. This very promising therapy resulted in an increase up to 14 points in the percentage of predicted FEV_1_ but also in significant improvements in sweat chloride concentration, pulmonary exacerbations and quality of life.

While there is variability in response, the targeted therapies are transforming the life of CF patients and their advent is expected to further improve patient survival in the coming years. As these molecules have only been marketed since 2012, it is too early to assess their impact on patient survival. This is why an American team has recently sought to model, through simulations based on data from the US CF Registry, the long-term health outcomes of CF patients treated with the lumacaftor/ivacaftor combination [95]. This treatment is predicted to increase the estimated median age of survival of p.Phe508del homozygous patients by 6.1 years. The increment in survival is further improved by the initiation of treatment at an early age and the persistence of treatment (an increment of 17.7 years if the treatment is started at age 6 and of 3.8 years if it is started at age 25). 

Over the past decade, the treatment of CF has therefore shifted from a therapy treating the symptoms to a therapy that also restores the function of the CFTR protein. These targeted therapies have expanded the field of personalized or precision medicine [89]. 

## 5. Conclusions

The epidemiological profile of CF has changed considerably in recent decades. This disease is no longer the most common serious illness in children but is now also a serious genetic disease among adults. Today, more than half of the patients are adults and patient survival has substantially increased with an estimated median age of survival close to 50 years today. The incidence of CF appears to be declining in most regions. The discovery of the *CFTR* gene in 1989 upset our knowledge of the pathophysiology of the disease. It has made it possible to better understand phenotypic variability through studies of genotype/phenotype correlations and has led to significant progress in the diagnosis and management of CF patients and their families. It also paved the way for pharmacology work and CFTR modulator therapies have been marketed for 10 years. Such treatments are revolutionizing the treatment of CF and transforming the life of CF patients. All these phenomena have contributed to changing the epidemiology of CF. The advent of targeted therapies is expected to further improve patient survival in the future. Efforts must nevertheless continue to find other efficient drugs, optimize treatment adherence and promote equitable access to these therapies.

## Figures and Tables

**Table 1:** Characteristics of the cystic fibrosis (CF) population and survival estimates presented in various CF registry annual data reports.

CF Registry [ref]		Year	Patients	Median Age	Age ≥ 18 y.	Age ≥ 40 y.	Median Age at Death	Median Age of Survival
			n	y.	%	%	y.	y.
**Australia**	[48]	2017	3151	19.6	53.7%	-	35.6	-
**Belgium**	[49]	2016	1275	22.5	61.2%	-	-	-
**Canada**	[10]	2018	4370	23.5	61.5%	15.9%	33.0	52.1
**ECFS**	[50]	2017	48204	18.5	51.3%	-	29.0	-
**France**	[51]	2017	7114	20.3	55.9%	11.9%	33.8	-
**Ireland**	[52]	2018	1239	20.9	58.5%	11.3%	33.0	44.4
**UK**	[11]	2018	10509	20.0	54.7%	-	32.0	47.3
**USA**	[12]	2018	30775	19.8	54.6%	-	30.8	47.4

This table only presents data from the CF registries for which at least two of the indicators of interest were available. ECFS: CF Registry of the European Cystic Fibrosis Society. y.: years.
